# MIBiG 2.0: a repository for biosynthetic gene clusters of known function

**DOI:** 10.1093/nar/gkz882

**Published:** 2019-10-15

**Authors:** Satria A Kautsar, Kai Blin, Simon Shaw, Jorge C Navarro-Muñoz, Barbara R Terlouw, Justin J J van der Hooft, Jeffrey A van Santen, Vittorio Tracanna, Hernando G Suarez Duran, Victòria Pascal Andreu, Nelly Selem-Mojica, Mohammad Alanjary, Serina L Robinson, George Lund, Samuel C Epstein, Ashley C Sisto, Louise K Charkoudian, Jérôme Collemare, Roger G Linington, Tilmann Weber, Marnix H Medema

**Affiliations:** 1 Bioinformatics Group, Wageningen University, Wageningen, NL, The Netherlands; 2 Novo Nordisk Foundation Center for Biosustainability, Technical University of Denmark, Lyngby, DK, The Netherlands; 3 Fungal Natural Products Group, Westerdijk Fungal Biodiversity Institute, Utrecht, NL, The Netherlands; 4 Department of Chemistry, Simon Fraser University, Burnaby, CA, USA; 5 Evolution of Metabolic Diversity Laboratory, Langebio, Cinvestav-IPN, Irapuato, MX, Mexico; 6 BioTechnology Institute, University of Minnesota-Twin Cities, MN, USA; 7 Biointeractions and Crop Protection, Rothamsted Research, Harpenden, UK; 8 Department of Chemistry, Haverford College, Haverford, PA, USA

## Abstract

Fueled by the explosion of (meta)genomic data, genome mining of specialized metabolites has become a major technology for drug discovery and studying microbiome ecology. In these efforts, computational tools like antiSMASH have played a central role through the analysis of Biosynthetic Gene Clusters (BGCs). Thousands of candidate BGCs from microbial genomes have been identified and stored in public databases. Interpreting the function and novelty of these predicted BGCs requires comparison with a well-documented set of BGCs of known function. The MIBiG (Minimum Information about a Biosynthetic Gene Cluster) Data Standard and Repository was established in 2015 to enable curation and storage of known BGCs. Here, we present MIBiG 2.0, which encompasses major updates to the schema, the data, and the online repository itself. Over the past five years, 851 new BGCs have been added. Additionally, we performed extensive manual data curation of all entries to improve the annotation quality of our repository. We also redesigned the data schema to ensure the compliance of future annotations. Finally, we improved the user experience by adding new features such as query searches and a statistics page, and enabled direct link-outs to chemical structure databases. The repository is accessible online at https://mibig.secondarymetabolites.org/.

## INTRODUCTION

Plants, microbes and fungi produce a large variety of specialized metabolites that are often uniquely found in one or a few species. From the dawn of civilization, humans have tapped into this treasure trove for medicinal, economic or recreational purposes. Within the last decade, genome-based discovery of specialized metabolites has become a widely adopted practice within both the scientific community and commercial settings. The magnitude of these efforts is continuously growing because of the ongoing increase in availability of genome and metagenome assemblies in public databases. These sequences can be mined for the presence of Biosynthetic Gene Clusters (BGCs): multi-enzyme loci that encode the biosynthetic pathways for one or more specific compounds.

Thousands of candidate BGCs have thus been identified using computational tools such as antiSMASH (1) and ClusterFinder (2). Databases like IMG-ABC (3) and antiSMASH-DB (4) store many thousands of such computationally predicted BGCs, potentially coding for a very diverse range of natural product classes. To unravel the function and novelty of current and future candidate BGCs, knowledge on previously characterized BGCs is essential. This calls for a standardized deposition and extraction of BGCs associated with molecules of known chemical structure, as this relevant knowledge is usually buried inside the text of scientific articles.

A first step to this end was taken in 2013, when ClusterMine360 (5) appeared, the first database of BGCs with known products, containing data on around 300 gene clusters. In 2015, the MIBiG (Minimum Information about a Biosynthetic Gene Cluster) Data Standard and Repository was established, containing 1170 BGC entries that were manually curated through a community effort, the results of which could be accessed via a fairly simple web application (6). Now, the MIBiG repository has become a central reference database for BGCs of known function, and provides the basis for comparative analyses in antiSMASH (1) via the KnownClusterBlast module. It has enabled many computational analyses of BGC function and novelty central to both small and large-scale studies of microbes and microbial communities. For example, Crits-Cristoph *et al.* (7) recently used MIBiG to assess and highlight the exceptional novelty of BGCs across 376 metagenome-assembled genomes of uncultivated soil bacteria from understudied phyla, by showing that most of these BGCs lacked any homology to gene clusters from MIBiG. Similarly, Bahram *et al.* (8) used homology searches against MIBiG to identify fungal BGCs associated with antibacterial activity across 7560 metagenomic samples, based on a set of MIBiG gene clusters whose products could be annotated with this activity; thus, they were able to show that the abundance of such ‘antibacterial’ BGCs correlated with the presence of antimicrobial resistance genes across soils. Yet another usage is illustrated by the ClusterCAD tool (9), which sources BGC data from MIBiG as a starting point for the computer-aided design of new biochemical pathways.

**Figure 1.**
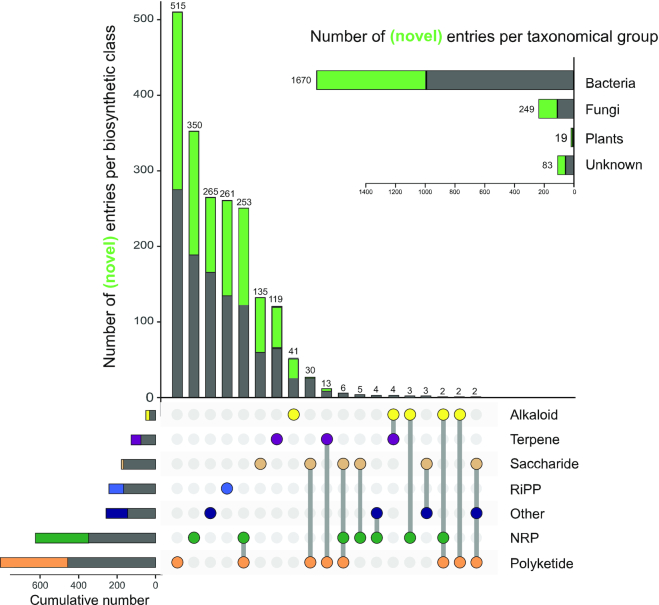
Distribution of taxonomic kingdoms and biosynthetic classes for all BGCs present in and added to MIBiG 2.0. Statistics are taken after the restructuring effort, and include retired entries. New entries are depicted in light green. Only (hybrid) classes comprising more than one BGC entry are listed in the figure. The intersection diagram is generated using the UpSetR tool (14).

Here, we provide an updated MIBiG version 2.0, which has been significantly expanded through the addition of 851 new entries over the past five years (Figure 1). Moreover, we performed extensive re-annotation of the entire database, increasing the overall data quality by improving the data schema, by adding hundreds of literature references and chemical structures and by providing cross-links to recently emerged databases of chemical structures and analytical data. Finally, we added useful functionalities to the online repository to make it more user-friendly, by enabling fast filtering based on compound names, taxonomic identifiers or biosynthetic classes, and facilitating the building of Boolean queries.

## METHODS AND IMPLEMENTATION

### Manual curation of entries

Since its inception in 2015, MIBiG has provided an online submission form for adding new entries. To submit a new entry, a user starts by requesting a MIBiG accession number. This is done through submitting the product name(s) and the sequence information of the BGC, preferably in the form of a set of coordinates corresponding to the BGC’s position within an NCBI Genbank accession. After the request is approved by MIBiG staff, the workflow subsequently provides an extended entry form where users can input more detailed information. This crowdsourcing, open-for-all approach has garnered 140 new entries since 2015, with contributions coming from various experts all over the world.

Because not all newly characterized BGCs are submitted to the database, we actively complemented this crowdsourcing approach by periodically organizing in-house ‘Annotathons’, where multiple scientists sat together for an entire day to work on MIBiG curation (Supplementary Table S1). This has yielded 702 new entries, and annotation quality improvements for over 600 BGCs.

More recently, we have introduced an additional MIBiG curation process into the classroom environment with the help of a comprehensive and very specific set of guidelines for the students (10,11). By giving one task to multiple students to work on independently, and later on having an expert (the teacher) to combine and validate the results, we have generated an additional 10 high quality BGC entries, for actinomycin, carbapanem, daptomycin, ebelactone, lipstatin, nocardicin A, obaflourin, oxazolomycin, salinosporamide and tabtoxin. Scaling up this process in the future may allow the annotations of many more important entries, which have remained incomplete, because, e.g. the scientists who have worked on the pathway are no longer active in the field.

### Data quality improvements

The MIBiG specification needs to capture the architectural and enzymatic variety present in currently described BGCs, and needs to stay flexible enough to also accommodate future discovery of even more diverse clusters and metabolites. In the initial MIBiG release in 2015, we relied only on the cluster submission form to aid annotators in creating valid entries. Now, we also adopted the JSON schema description and validation technology (https://json-schema.org) that was recently made available, which enables us to embed validation and dependency rules into the schema. This can then be processed programmatically via libraries implemented in almost all popular programming languages.

After implementing the JSON schema updates, we performed a thorough data quality assessment of the entire repository, fixing empty or mistyped information in the data, removing duplicate entries, adding and correcting structural information, adding new entries, and retiring entries we deemed of insufficient quality, e.g. when the sequence assembly does not cover the full DNA sequences of the cluster region, effectively removing spatial context from the BGC data (Supplementary Table S2).

Finally, additional cross-links have been established with the Natural Products Atlas (https://www.npatlas.org/) and the GNPS spectral library (12). This enables users to acquire information about specialized metabolites with structures similar to those found in MIBiG, and to identify mass spectra linked to a specific molecule of interest. These additions further complement the already existing links with PubChem (13) and other compound databases. Connections were made according to compound names and structures matching between the annotated BGCs and the chemical databases.

### The new database architecture

Previously stored in a collection of static HTML pages, the MIBiG data has now been migrated into a relational database. This setup allows users to query the metadata, using either a simple search form or an interactive query builder that assists in building more complex queries. A REST-like web API (https://github.com/mibig-secmet/mibig-api/) handles access to the underlying PostgreSQL (https://www.postgresql.org/) database. A single-page web application written in AngularJS (https://angularjs.org/) runs the user interface allowing users to browse a repository overview, view statistics about the clusters in the database, or run metadata queries. The individual BGC pages are generated using a customised antiSMASH 5 module that sideloads a MIBiG annotation file (in JSON format). Annotations generated by antiSMASH are also produced alongside the manually curated MIBiG information.

## RESULTS AND DISCUSSION

### Data overview

#### BGC diversity

The MIBiG repository version 2.0 encompasses 2021 manually curated BGCs with known functions, which is a 73% increase from the original 1170. Categorically, there are seven structure-based classes: ‘Alkaloid’, ‘Nonribosomal Peptide (NRP)’, ‘Polyketide’, ‘Ribosomally synthesised and Post-translationally modified Peptide (RiPP)’, ‘Saccharide’, ‘Terpene’, and ‘Other’. These classes may overlap, as in the case of Polyketide-NRP hybrids such as Rapamycin (BGC0001040) and Bleomycin (BGC0000963). The ‘Other’ category includes cyclitols like cetoniacytone A (BGC0000283), indolocarbazoles like rebeccamycin (BGC0000821) and phosphonates like fosfomycin (BGC0000938). MIBiG is currently mostly populated with entries of the Polyketide (825 BGCs) and NRP (627 BGCs) classes. Hybrids of these classes are also prominently featured. Proportionally, the new entries also contain a lot of Polyketides and NRPs, together comprising more than half (59%) of the batch. Taxonomically, BGCs in MIBiG have mostly bacterial or fungal origins (in particular, the genus *Streptomyces* is the most prominent with 568 BGCs, followed by *Aspergillus* at 79 and *Pseudomonas* at 61), with only 19 coming from plants.

#### Annotation completeness

BGCs in MIBiG start with a ‘minimal’ annotation, meaning that it consists only of locus information (Genbank accession and coordinates of the cluster), a compound name, and at least one reference publication. Detailed information such as compound structures (stored as a SMILES string), class-specific attributes (e.g. Polyketide synthase (PKS) modules), are usually, but not always, present. Prior to the schema restructuring, there were 2021 BGCs, of which 770 did not have any chemical structure of their product(s) associated with them, and 500 had missing or incomplete properties. With the results of all manual re-curation efforts compiled into the dataset, we have incorporated new structure information for 220 BGCs, solved most of the issues with incomplete properties, and retired some BGCs of low annotation quality (Supplementary Table S2). (These retired entries are still available for download.) An overview of the updates is shown in Table 1.

**Table 1.** Annotation completeness of BGCs in MIBiG 2.0 before and after the restructuring effort

	Before	After
**Entries without structure information**	**770**	**550**
**Entries with incomplete properties**	**500**	**18**
• No reference publication	148	11
• Values unknown to the schema	235	0
• Others	158	7
**Retired entries**		**105**
• Duplicate BGC		11
• Poor sequence quality		70
• Poor annotation quality		24

### A new online repository

**Figure 2.**
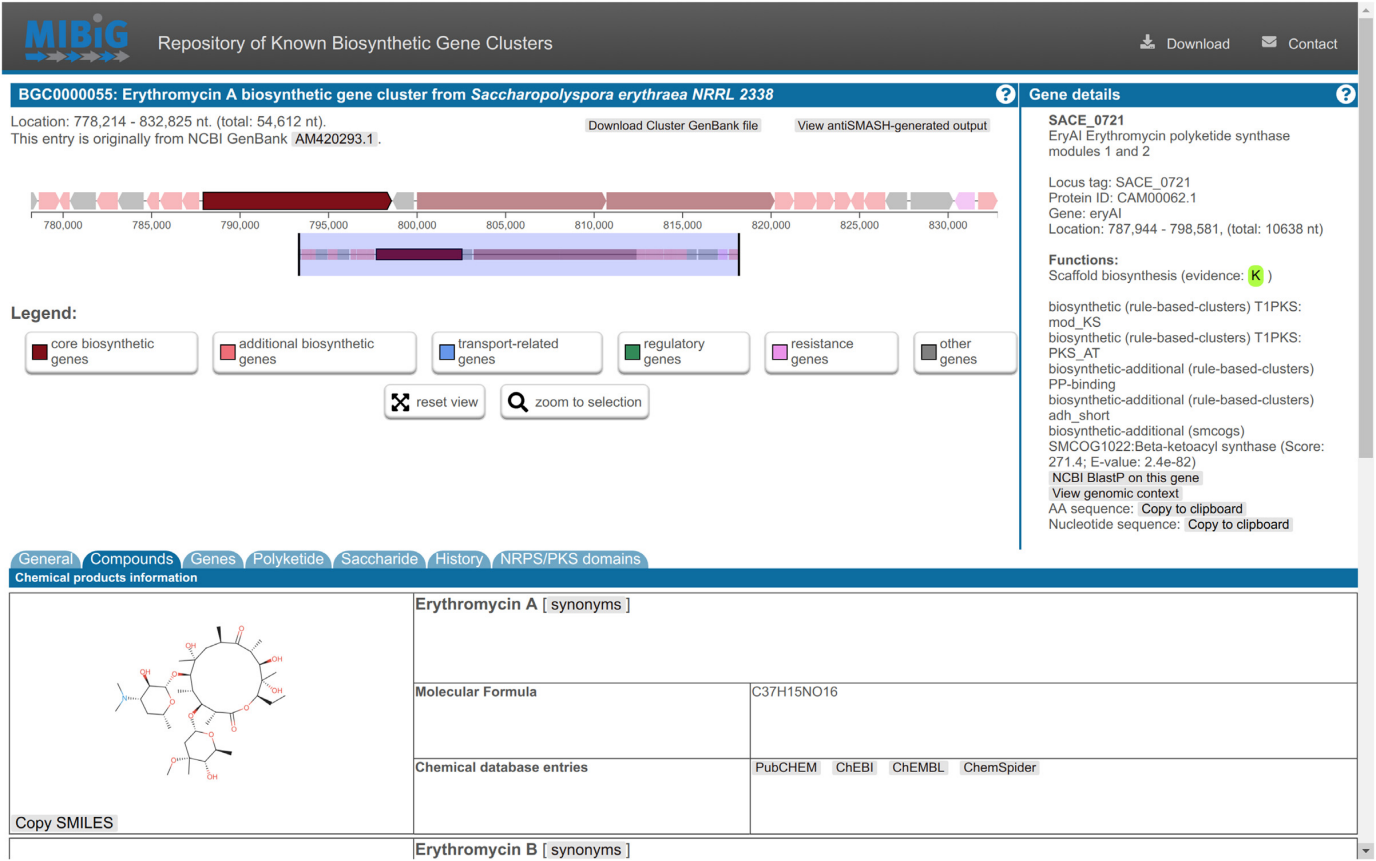
The new per-BGC overview page. The locus overview (top-left) section allows panning, zooming, or highlighting specific genes, for which the information would be displayed in the gene details (top-right) section. In the lower section, the ‘Compounds’ tab is currently selected, showing all compound-related information of the BGC, such as chemical structure, molecular formula, or linked databases. Other data is linked to other specific tabs.

The overall design of the old repository has been thoroughly refreshed. Rows in the ‘Repository’ page can now be filtered and sorted based on annotation metadata, such as species names or biosynthetic classes. The BGC page itself takes advantage of the modernized, well-organized look of antiSMASH 5 (1). Annotation data are now organized into their own category tabs, e.g. ‘General’, ‘Compounds’, ‘History’, ‘Polyketide’, ‘NRPS’ and so on (Figure 2). Some new functionalities were also introduced to the main page. ‘Statistics’ displays a real-time overview of the database, such as compound class distribution, taxonomy, and annotation completeness. ‘Search’ provides users the ability to build complex queries based on MIBiG metadata, for example ‘find all complete RiPP BGCs from the genus *Streptomyces*’.

## DATA AVAILABILITY

The MIBiG Repository is available at https://mibig.secondarymetabolites.org/. There is no access restriction for academic or commercial use of the repository and its data. The source code components, JSON-formatted data standard, and SQL schema for the MIBiG Repository are available on GitHub (https://github.com/mibig-secmet) under an OSI-approved Open Source license.

## Supplementary Material

gkz882_Supplemental_Files
